# Isolation of plant-derived exosome-like nanoparticles (PDENs) from *Solanum nigrum* L. berries and Their Effect on interleukin-6 expression as a potential anti-inflammatory agent

**DOI:** 10.1371/journal.pone.0296259

**Published:** 2024-01-04

**Authors:** Natasya Emmanuela, Daisy Ramadhani Muhammad, Iriawati, Christofora Hanny Wijaya, Yuliana Maria Diah Ratnadewi, Hiroshi Takemori, Ika Dewi Ana, Ratna Yuniati, Windri Handayani, Triati Dewi Kencana Wungu, Yasuhiko Tabata, Anggraini Barlian

**Affiliations:** 1 School of Life Sciences and Technology, Institut Teknologi Bandung, Bandung, Indonesia; 2 Department of Food Science and Technology, Institut Pertanian Bogor, Bandung, Indonesia; 3 Faculty of Mathematics and Natural Sciences, Institut Pertanian Bogor, Bandung, Indonesia; 4 Department of Chemistry and Biomolecular Science, Gifu University, Gifu, Japan; 5 Faculty of Dentistry, Universitas Gadjah Mada, Yogyakarta, Indonesia; 6 Faculty of Mathematics and Natural Sciences, Universitas Indonesia, Depok, Indonesia; 7 Faculty of Mathematics and Natural Sciences, Institut Teknologi Bandung, Bandung, Indonesia; 8 Department of Regeneration Science and Engineering Institute for Life and Medical Science (LiMe), Kyoto University, Kyoto, Japan; 9 Research Center of Nanoscience and Nanotechnology, Institut Teknologi Bandung, Bandung, Indonesia; Pakistan Council of Scientific and Industrial Research, PAKISTAN

## Abstract

Inflammation is a temporary response of the immune system that can be treated using common anti-inflammatory drugs. However, prolonged use of these drugs increases the risk of adverse side effects. Accordingly, there is an increasing need for alternative treatments for inflammation with fewer side effects. Exosomes are extracellular vesicles secreted by most eukaryotic cells and have been studied as a candidate for cell-free therapy for inflammatory diseases due to their immunomodulatory and anti-inflammatory properties. In recent years, the focus of exosome research has shifted from animal cell-derived exosomes to plant-derived exosome-like nanoparticles (PDENs). Plant-derived exosome-like nanoparticles (PDENs) are easier to obtain, have minimal safety concerns, and can be produced in higher quantities and lower cost than exosomes derived from animal cells. In this study, the isolation and analysis of the anti-inflammatory potential of PDENs from black nightshade berries (*Solanum nigrum* L.) were carried out. The results of isolation and characterization showed that PDENs had a spherical morphology, measuring around 107 nm with zeta potential of -0.6 mV, and had a protein concentration of 275.38 μg/mL. PDENs were also shown to be internalized by RAW264.7 macrophage cell line after 2 hours of incubation and had no cytotoxicity effect up to the concentration of 2.5 μg/mL. Furthermore, exposure to several doses of PDENs to the LPS-stimulated RAW264.7 cell significantly decreased the expression of pro-inflammatory cytokine gene IL-6, as well as the expression of IL-6 protein up to 97,28%. GC-MS analysis showed the presence of neral, a monoterpene compound with known anti-inflammatory properties, which may contribute to the anti-inflammatory activity of PDENs isolated from *Solanum nigrum* L. berries. Taken together, the present study was the first to isolate and characterize PDENs from *Solanum nigrum* L. berries. The results of this study also demonstrated the anti-inflammatory activity of PDEN by suppressing the production of IL-6 in LPS-stimulated RAW264.7 cells.

## Introduction

Inflammation is a temporary response of the immune system that acts as a defense mechanism against pathogens or other stimuli such as tissue injury [1]. In some cases, inflammation can persist for a longer period leading to chronic disease. Chronic inflammation contributes to pathogenesis and mortality of several diseases, including diabetes, autoimmune diseases, obesity, chronic respiratory diseases, and cancer [2]. In general, inflammation can be effectively treated using common anti-inflammatory drugs, such as corticosteroids or non-steroidal anti-inflammatory drugs (NSAIDs). However, the prolonged use of corticosteroids or NSAIDs is associated with adverse side effects, including infection, diabetes, osteoporosis, stomach ulcers, kidney failure, stroke, heart failure, and hypertension, particularly in older adults [3,4]. Accordingly, there is an increasing need for alternative pharmacological agents for the treatment of inflammation with fewer effects.

Exosomes are extracellular vesicles secreted by most eukaryotic cells with a diameter ranging from 30 to 160 nm. Exosomes are known to mediate intercellular communication by modulating the biological function of target cells through specific signaling pathways. Exosomes contain a variety of molecules, including proteins, lipids, and nucleic acids [5]. In regenerative medicine, exosomes have been studied as a potential cell-free therapy with specific cell-targeting properties, fewer safety concerns, and lower cost of manufacturing compared to cell-based therapy [6]. Exosomes may also have utility in the treatment of inflammatory diseases due to their immunomodulatory and anti-inflammatory properties [7,8].

Although many previous studies have focused on exosomes derived from mammalian cells, there has recently been increasing interest in plant-derived exosome-like nanoparticles (PDENs). PDENs have similar properties, morphology, and size distribution to exosomes derived from mammalian cells [9]. PDENs are easier to obtain due to the abundance of plant sources, have minimal safety concerns, and can be produced in higher quantities and lower cost than exosomes derived from mammalian cells [9]. PDENs can also regulate gene expression in a cross-kingdom manner [10]. Moreover, several species of plants have been shown to produce PDENs with anti-inflammatory properties, such as ginger [11], cabbage [12], onion [13], and lemon [14]. These findings further confirm the potential utility of PDENs as an alternative treatment for inflammation that can overcome the disadvantages associated with common anti-inflammatory drugs and exosomes derived from mammalian cells. However, there are limited reported studies on PDENs and many plant sources of PDEN have yet to be investigated.

*Solanum nigrum* L., commonly known as black nightshade and locally known as leunca, is a widely cultivated and consumed plant in Indonesia. *Solanum nigrum* L. has been shown to have antioxidant, antimicrobial, antipyretic, anticancer, hepatoprotective, immunomodulatory, and anti-inflammatory properties [15,16]. *In vitro* and *in vivo* studies have demonstrated methanolic and steroidal saponin compounds extracted from the berries of *Solanum nigrum* L. have anti-inflammatory properties [17,18]. Despite these broad medicinal properties, the anti-inflammatory effect of PDENs derived from *Solanum nigrum* L. berries has not previously been studied. Accordingly, the present study aimed to investigate the anti-inflammatory properties of PDENs derived from *Solanum nigrum* L. berries *in vitro*.

## Materials & methods

### Materials and cell culture

RAW264.7 cells (a murine macrophage cell line; Elabscience) were cultured in Dulbecco’s modified Eagle’s medium (DMEM; Sigma) supplemented with 10% (v/v) fetal bovine serum (Gibco) and 1% antibiotic-antimycotic (Gibco). Cells were grown at 37°C under 5% CO_2_.

### Isolation of PDENs from *Solanum nigrum* L. berries

Fresh black nightshade (*Solanum nigrum* L.) berries were purchased from a local market in Bandung, Indonesia. PDENs were isolated according to the method of Kalarikkal *et al*. (2020) [19] with slight modifications. First, fresh berries were washed with tap water and ground using a food chopper. Next, 40 grams of juice were filtered using a 40 μm and 100 μm nylon filter. The filtered juice was sequentially centrifuged at 2,000 × g for 10 min, 6,000 × g for 20 min, and 10,000 × g for 40 min to remove large particles and cell debris. The supernatant obtained from the final centrifugation step was added to polyethylene glycol 6000 (Himedia) to a final concentration of 5% (w/v) and then stored at 4°C overnight. The next day, the supernatant was centrifuged at 8000 × g for 30 min to precipitate PDENs. The pellet formed by centrifugation was then dissolved in 10 mL ddH_2_O, followed by filtration using a polyethersulfone (PES) syringe filter with pore sizes of 0.45 μm and 0.22 μm (Minisart®, Sartorius). The filtered PDEN solution was then stored at -20°C.

### Characterization of PDENs

The morphology of PDENs was characterized by a transmission electron microscope (HT7700, 120 kV) using the negative staining method. Nanoparticle tracking analysis (NTA) was performed using ViewSizer 3000 (Horiba) to evaluate the size distribution and concentration of PDENs. Dynamic light scattering (DLS) analysis was performed using a particle size analyzer (Horiba SZ-100i) to measure the zeta potential of PDENs. The total protein concentration of PDENs was measured using PierceTM BCA Protein Assay kits (Thermo Fisher) according to the manufacturer’s protocol.

### Storage temperature and duration of PDENs

To assess the effect of storage temperature and duration on PDENs stability, fresh PDENs were stored at 4°C and -20°C for four weeks. The particle size of PDENs was measured at week 0–4 using DLS analysis.

### Intracellular uptake of PDENs

PDENs were stained using PKH67 Green Fluorescent Cell Linker kits (Merck) according to the protocol provided by the supplier. First, 50 μL aliquots of the PDEN samples were mixed with 500 μL of diluent C. Then, 500 μL of diluent C was also mixed with 2 μL of PKH67. These two solutions (PDENs and PKH67) were mixed and incubated at room temperature for 4 minutes. The mixture was then added to 1 mL of 1% bovine serum albumin (BSA) and incubated for 1 minute. The final mixture was centrifuged at 4000 × g for 15 minutes. The pellet was collected and washed three times with phosphate buffer saline (PBS) followed by high-speed centrifugation. The pellet obtained from the final centrifugation step was dissolved in DMEM containing 1% antibiotic-antimycotic solution (Gibco).

RAW264.7 cells were grown on a coverslip of a six-well plate for two to three days. Culture media were then removed, and cells were washed three times using PBS. Cells then were incubated with varying concentration of PDENs at 37°C under 5% CO_2_ for two hours or six hours in duplicate. At the end of the incubation period, cells were fixed in 4% paraformaldehyde for 10 minutes. Cells were then washed using PBS and dehydrated using acetone at -20°C for 5 minutes. Nuclear staining with 42,6-diamidino-2-phenylindol (DAPI) was then performed. The cellular uptake of PDENs was observed using a confocal laser scanning microscope (Olympus FV-1200).

### Cell cytotoxicity assay

The cytotoxicity of PDENs to macrophage cell line was examined by MTT Assay. RAW264.7 cells were seeded at a density of 1 × 104 cells/well in 96-well plates and cultured for 24 hours. Cells were then treated with different doses of PDENs for 24 hours. The absorbance at 595 nm was measured using iMarkTM microplate absorbance reader (Bio Rad).

### Real-Time PCR analysis of interleukin-6 (IL-6) gene expression

RAW264.7 cells were seeded in six-well plates at a density of 2 × 105 cells/well and cultured for 24 hours. Cells were then pre-treated for 24 hours with 0.5 μg/mL, 1 μg/mL, and 2.5 μg/mL PDENs or 1 μg/mL dexamethasone (Sigma-Aldrich) in DMEM supplemented with 5% FBS. After 24 hours, cells were then stimulated with 10 ng/mL of Lipopolysaccharides/LPS (Sigma-Aldrich) for six hours to induce inflammation. Total RNA was isolated using *Quick*-RNA^TM^ Miniprep Plus Kit (Zymo Research).

Reverse transcription of the RNA to cDNA was performed using GoScript^TM^ Reverse Transcriptase (Promega). Then the cDNA was used for quantitative real-time PCR analysis using GoTaq® qPCR Master Mix. The expression of mouse GAPDH was used as internal control to standardize the data. Relative changes in gene expression were estimated using the 2^(−ΔΔCt)^ method.

### Enzyme-linked immunosorbent assay for interleukin-6 (IL-6)

RAW264.7 cells were seeded at a density of 3.8 × 104 cells in 24-well plates and cultured for 24 hours. Cells were then pre-treated for 24 hours with 0.1 μg/mL, 0.5 μg/mL, 1 μg/mL, and 2.5 μg/mL PDENs or 1 μg/mL dexamethasone (Sigma-Aldrich) in DMEM supplemented with 5% FBS. After 24 hours, cells were then stimulated with 10 ng/mL of LPS for six hours to induce inflammation. This experiment had three controls: negative control (serum-free DMEM), positive control (LPS treatment), inhibition control (LPS and dexamethasone). The concentration of IL-6 was quantified using mouse IL-6 ELISA kits (Elabscience®) according to the protocol provided by the supplier to measure pro-inflammatory cytokine secretion. The absorbance of each well at 450 nm was measured using an iMarkTM microplate absorbance reader (Bio Rad).

### GC-MS analysis of PDENs

The content of PDENs isolated from *S*. *nigrum* was analyzed by gas chromatography-mass spectrometry (GC-MS; Agilent) using DB-5 MS capillary column (30 m × 0.25 mm × 0.25 μm, Agilent). The initial oven temperature was set to 60°C, followed by an increment of 10°C/min up to 240°C. Helium was used as a carrier gas at constant pressure of 65 kPa. The injection system was carried out using split mode with split ratio of 1:25. The extract was dissolved in hexane (1:10) and injected into the column with the volume of 1 μL.

### Statistical analysis

The results are reported as mean ± standard deviation (SD), n ≥ 3. Data were analyzed using one-way ANOVA, with a *p-*value < 0.05 was considered statistically significant. Statistical analysis was performed using GraphPad Prism software (GraphPad Software, Inc.).

## Results

### Isolation and characterization of PDENs

**Fig 1:**
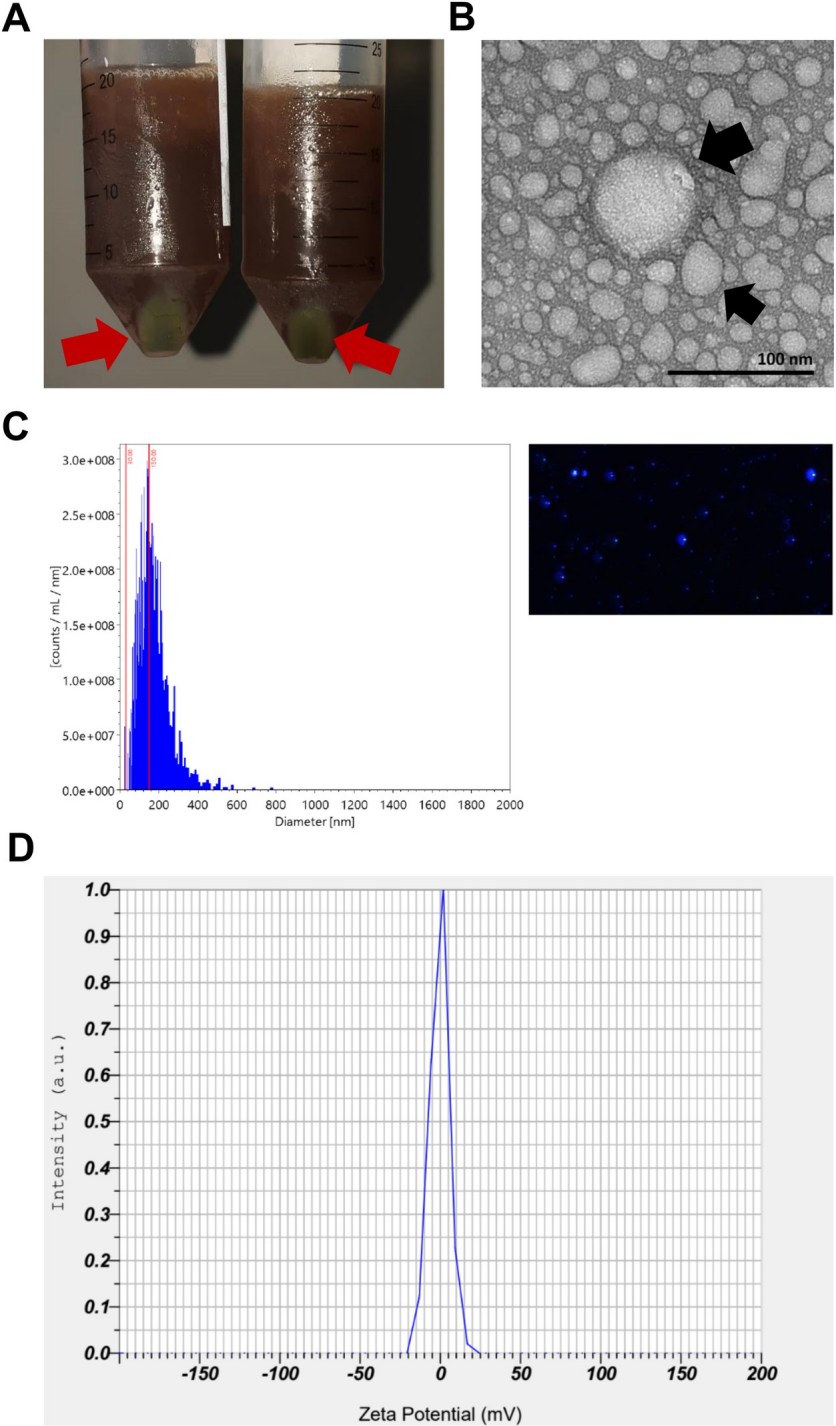
Characterization of PDENs isolated from *Solanum nigrum* L. berries. (A) PDENs in the form of green pellets (red arrows) after PEG precipitation, followed by centrifugation. (B) Morphology of PDENs by TEM imaging (black arrows); scale bar indicates 100 nm. (C) Size distribution & concentration of PDENs analyzed by NTA, and (D) Zeta potential of PDENs measured by DLS analysis.

The isolation of PDENs from *Solanum nigrum* L. berries was performed using PEG-based precipitation, which produced green pellets (**Fig 1A**). After isolation, the morphology of PDENs was analyzed using transmission electron microscopy (TEM). PDENs had a spherical morphology with a diameter ranging from 30 to 100 nm (**Fig 1B**). NTA results showed that the isolated PDENs had an average size of 107 nm, with a modal size of 139 nm and concentration of 8.1 × 10^9^ particles/mL (**Fig 1C)** within the integration range of 30–150 nm. DLS analysis demonstrated PDENs had an average zeta potential of -0.6 mV (**Fig 1D**). The BCA assay demonstrated 275.38 μg/mL of PDENs were isolated from *Solanum nigrum* L. berries.

### Uptake of PDENs by RAW264.7 cells

**Fig 2:**
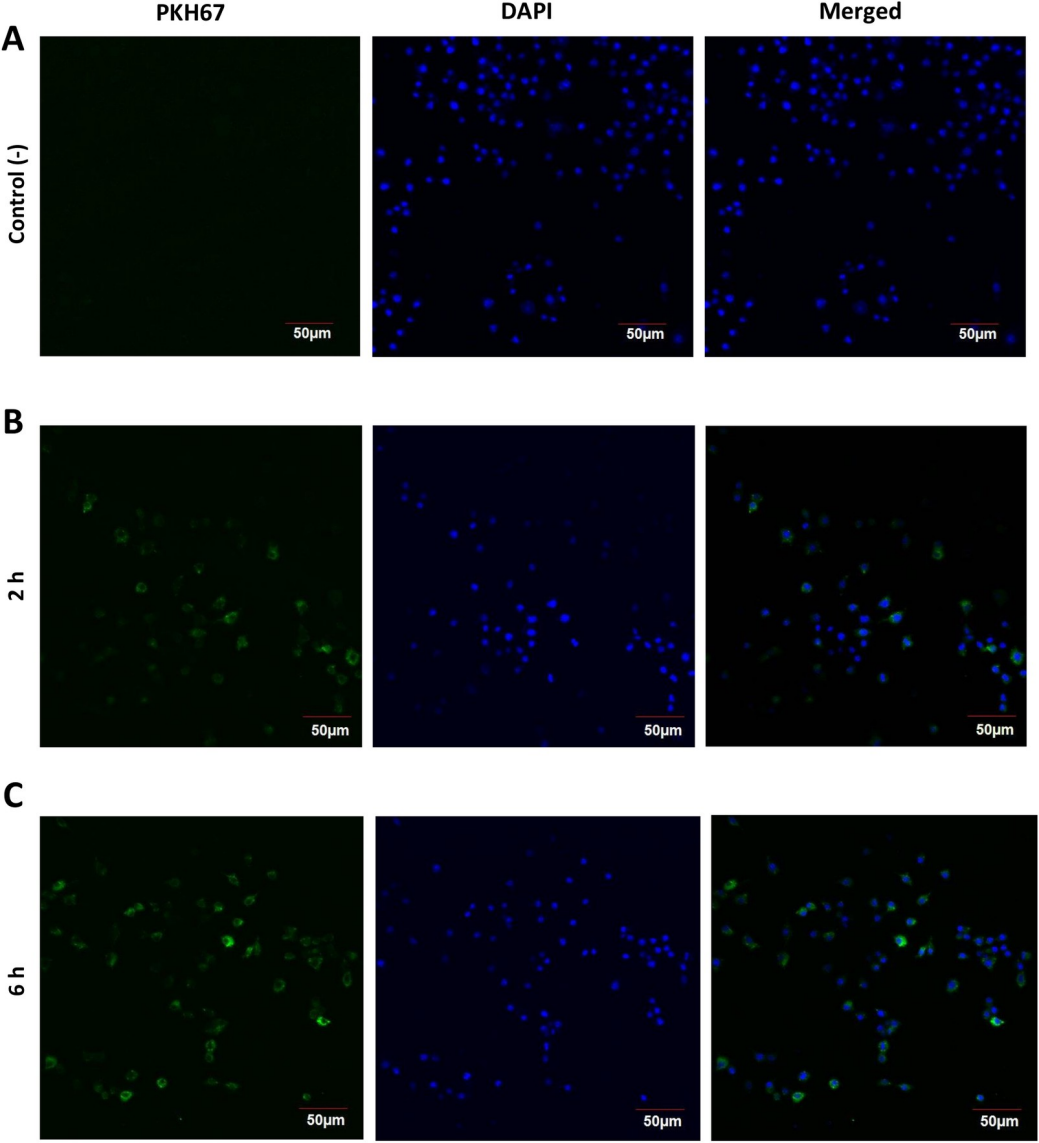
PDENs uptake by RAW264.7 macrophage cells. (Left) PKH67-labelled PDENs uptake by cells indicated by green color, (middle) cell nucleus stained with DAPI indicated by blue color, (right) merged images of PKH67-labelled PDENs and DAPI-stained nucleus. (A) Cells without PDENs treatment (negative control). (B) Cells treated with PDENs within 2 h of incubation time. (C) Cells treated with PDENs within 6 h of incubation time.

RAW264.7 cells were incubated with PDENs labeled with PKH67, a lipophilic green fluorescence dye, with the uptake of PDENs by RAW264.7 cells measured at two and six hours (**Fig 2**). Cell nuclei were stained with DAPI (blue). After two hours incubation, PDENs (green) were observed in the cytoplasm of RAW264.7. A stronger fluorescence signal in the cytoplasm indicated greater uptake of PDENs after incubation for six hours.

### Effect of storage temperature on PDENs

**Fig 3:**
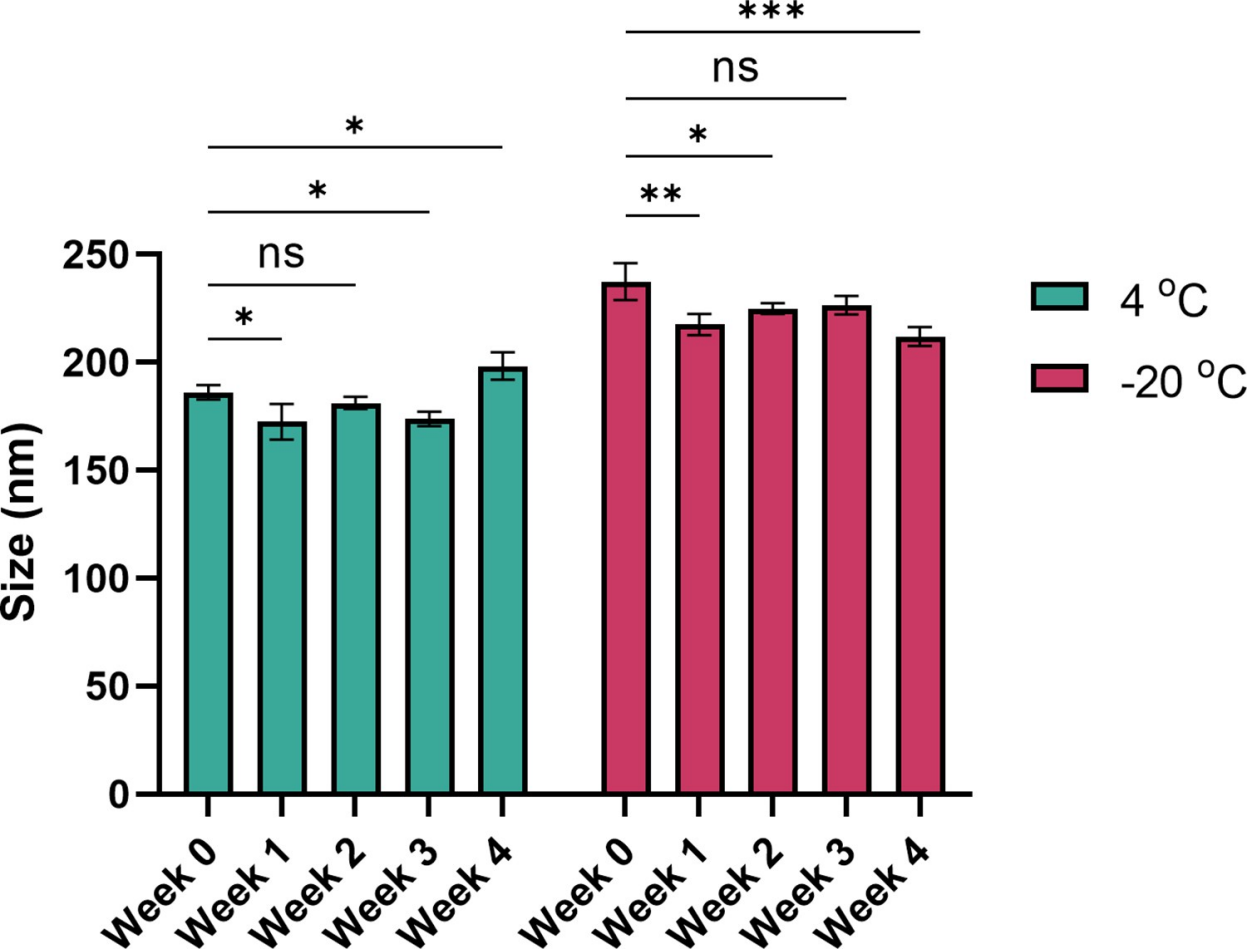
Effect of storage temperature and duration on the stability of PDENs. PDENs were stored at 4°C and -20°C for four weeks. The average size of PDENs was measured every week using DLS analysis. Data presented as mean ± standard deviation, (*p < 0.05, **p < 0.01, ***p < 0.001).

PDENs were stored at 4°C and -20°C for four weeks. The average size of PDENs was significantly decreased after a week of storage, both at 4°C and -20°C (**Fig 3**). However, there was a significant increase of particle size after four weeks of storage at 4°C.

### Effect of PDENs on cytotoxicity in RAW264.7 cells

**Fig 4:**
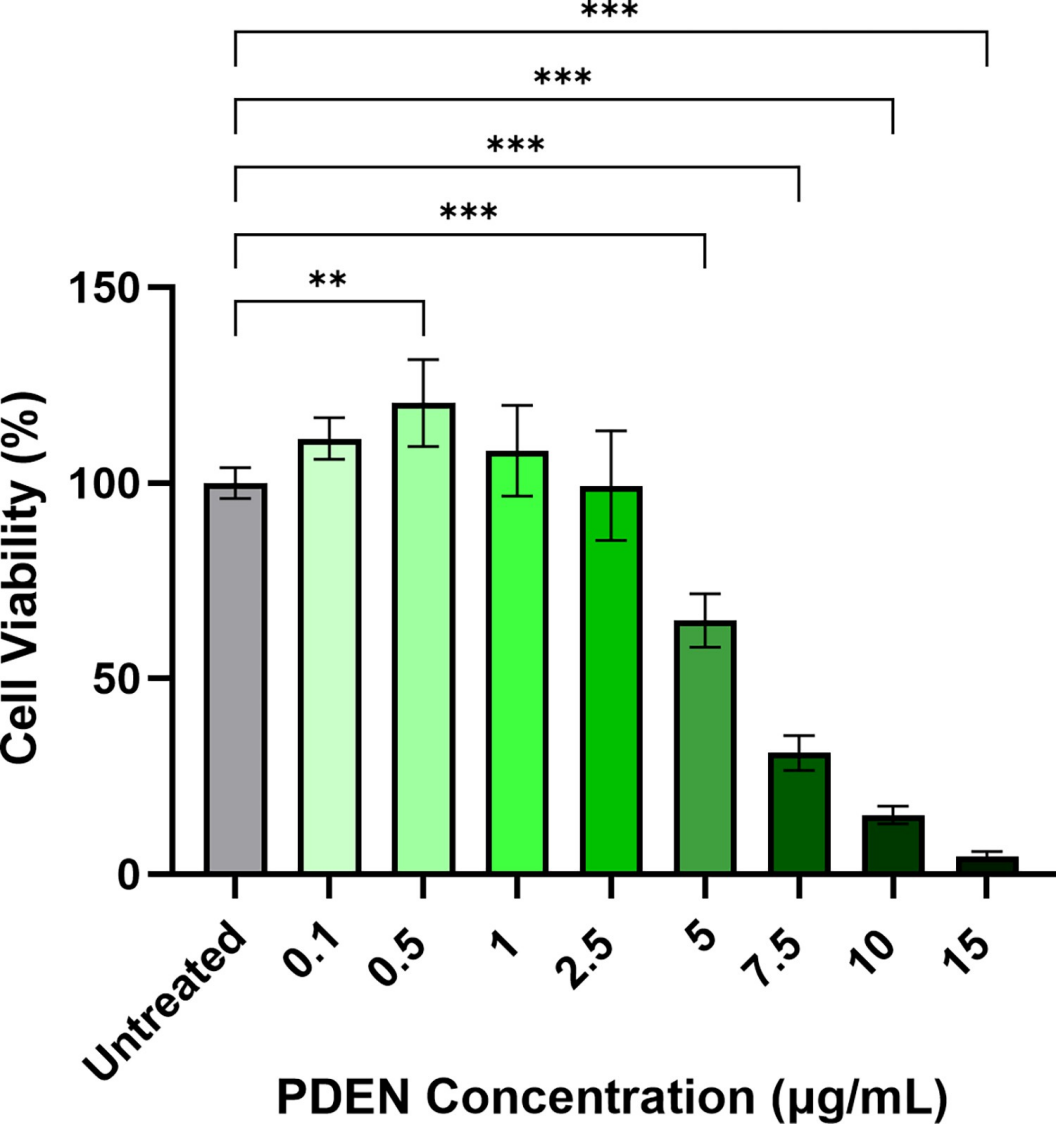
The cytotoxicity of PDENs in RAW264.7 cells examined by MTT assay. RAW264.7 Cells were treated with increasing concentrations of PDENs (0.1, 0.5, 1, 2.5, 5, 7.5, 10, and 15 μg/mL) for 24 h. Data presented as mean ± standard deviation, (*p < 0.05, **p < 0.01, ***p < 0.001, n = 5).

To assess the cytotoxicity of PDENs, RAW264.7 cells were treated with different concentrations of PDENs (0.1, 0.5, 1, 2.5, 5, 7.5, 10, and 15 μg/mL), then the cell viability was examined after 24 hours by MTT Assay. PDENs had no significant cytotoxic effects at concentrations up to 2.5 μg/mL compared to control untreated cells (**Fig 4**). Furthermore, the cell viability was significantly increased up to 120% when treated with 0.5 μg/mL of PDENs. However, cells treated with higher concentrations of PDENs (≥ 5 μg/mL) showed a significant decrease in viability to as low as 4%. Therefore, PDENs with concentrations above 2.5 μg/mL were not considered for further experiments.

### Anti-inflammatory effect of PDENs on LPS-stimulated RAW264.7 cells

To investigate the *in vitro* anti-inflammatory effect of PDENs, RAW264.7 cells were supplemented with different concentrations of PDENs (0.5, 1, and 2.5 μg/mL) or 1 μg/mL dexamethasone (DEX) for 24 hours, followed by LPS treatment for 6 hours to induce inflammation. Cells treated with LPS only (without PDENs) served as a positive control. For the inhibition control group, cells were treated with LPS and 1 μg/mL dexamethasone to compare the anti-inflammatory effect of PDENs and a commercial anti-inflammatory drug. The expression of pro-inflammatory cytokine gene IL-6 was analyzed using RT-qPCR and the amount of protein secreted by cells was measured using ELISA.

**Fig 5:**
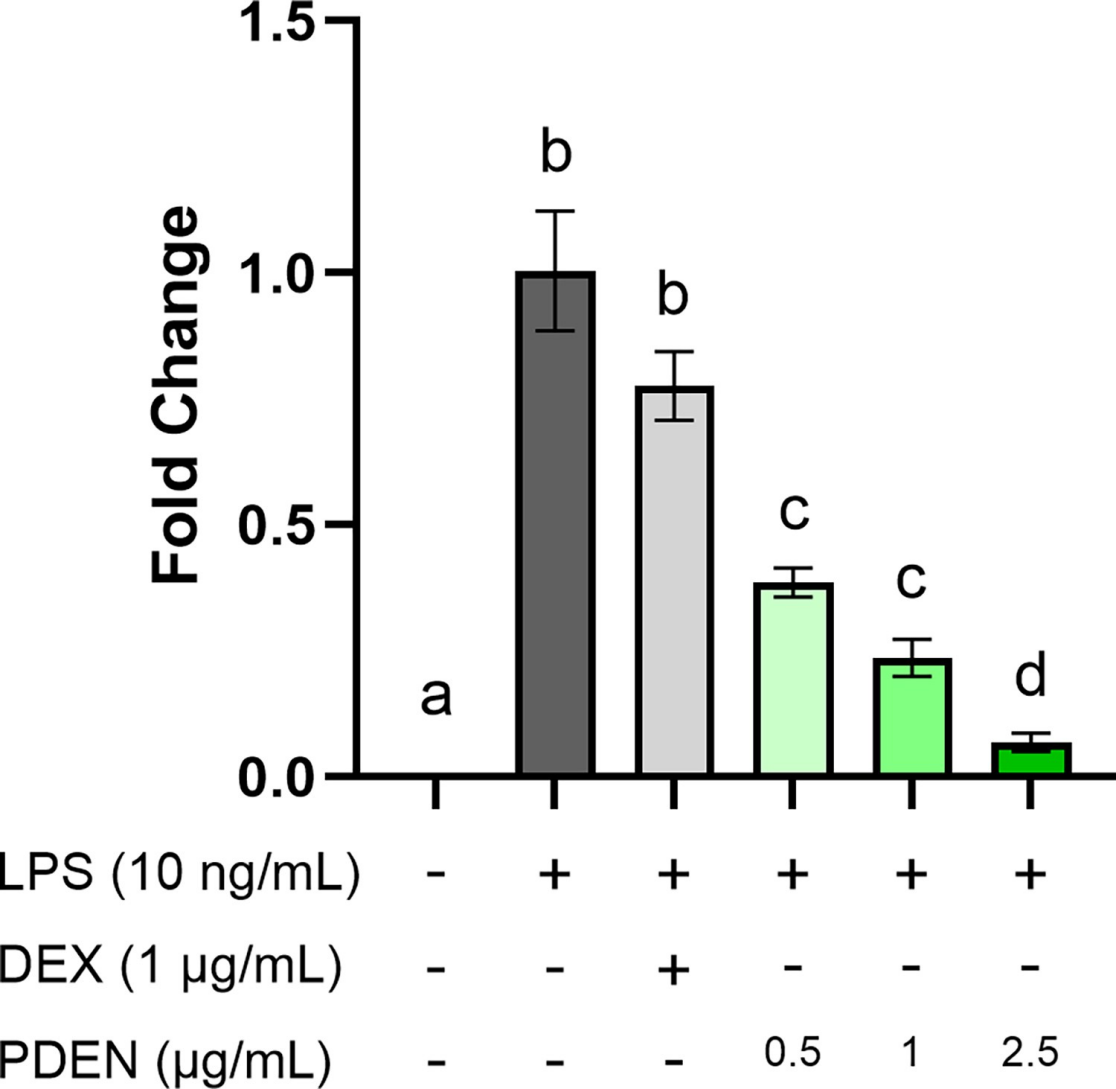
RT-PCR analysis of IL-6 gene expression in RAW264.7 pre-treated with PDENs. RAW264.7 cells were pre-treated with different concentrations of PDENs (0.5, 1, and 2.5 μg/mL), and 1 μg/mL dexamethasone (DEX) was used as an inhibition control. Data were calibrated against LPS treatment and presented as mean ± standard deviation. Different letters (a-d) above the bars indicate different significance group (p < 0.05, Tukey, n = 3).

**Fig 6:**
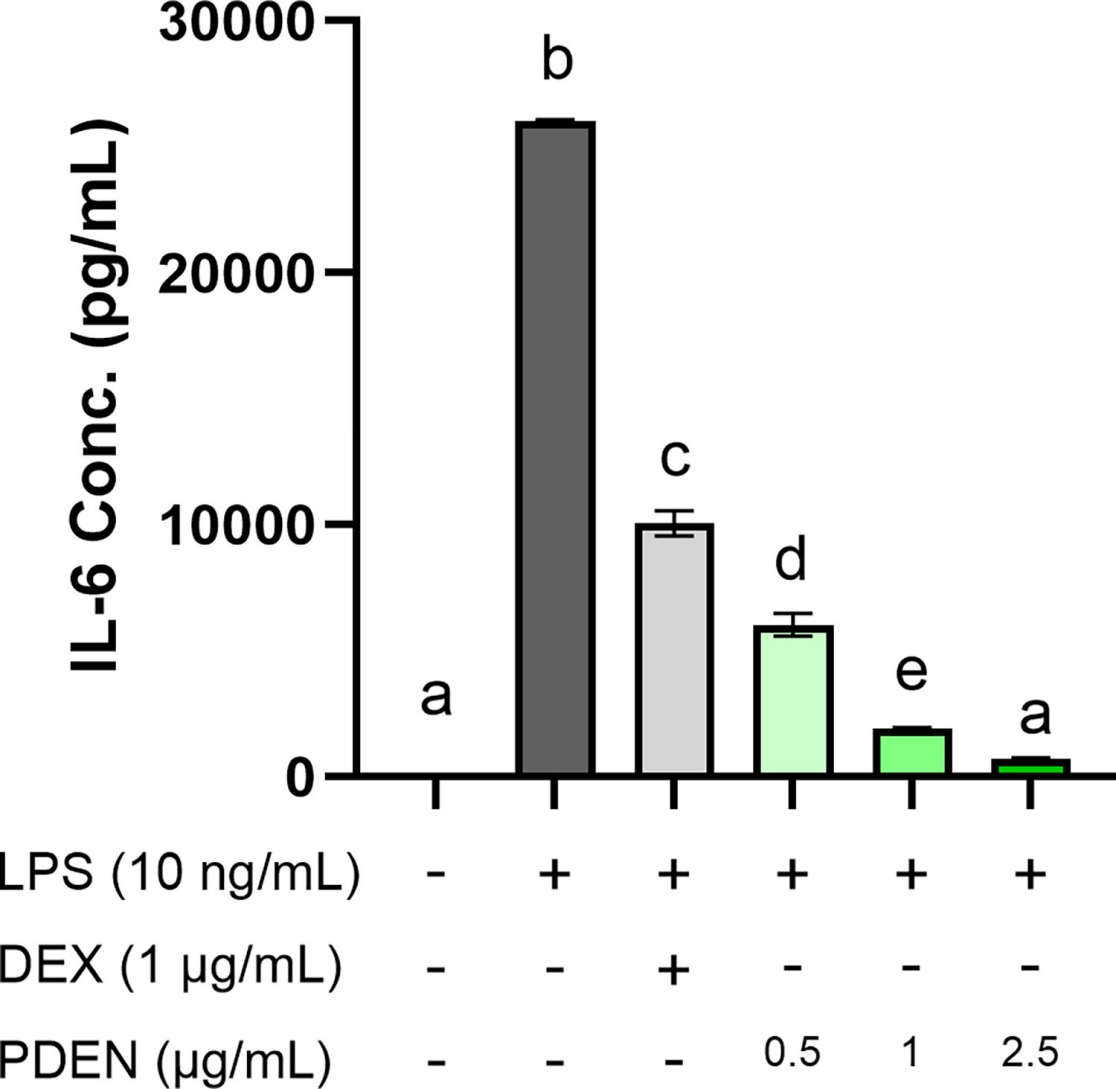
ELISA analysis of IL-6 secretion in RAW264.7 pre-treated with PDENs. RAW264.7 cells were pre-treated with different concentrations of PDENs (0.5, 1, and 2.5 μg/mL), and 1 μg/mL dexamethasone (DEX) was used as an inhibition control. Data were presented as mean ± standard deviation. Different letters (a-e) above the bars indicate different significance group (p < 0.05, Tukey, n = 3).

IL-6 gene expression was significantly downregulated in cells treated with different concentrations of PDENs compared with LPS-stimulated cells (**Fig 5**). PDENs with a concentration of 2.5 μg/mL had the best effect in downregulating the expression of pro-inflammatory cytokines IL-6. In addition, the expression of IL-6 cytokine was significantly lower in cells treated with PDENs at a concentration of 1 μg/mL compared to DEX-treated cells at the same concentration. These results were also confirmed at the protein level based on ELISA assay. Increased IL-6 cytokine secretion was observed in the positive control group compared to the untreated control and inhibition control groups (**Fig 6**). The secretion of IL-6 was lower in LPS-stimulated cells treated with PDENs in a dose-dependent manner. PDENs treatments with concentration of 0.5, 1, and 2.5 μg/mL significantly reduced the secretion of IL-6 levels by 76.82%, 92.92%, and 97.28% respectively, compared to the positive control group. Moreover, PDENs treatment with concentration of 1 μg/mL also showed to significantly downregulate IL-6 protein secretion levels by 81.70% compared to DEX-treated cells with the same concentration.

### Content analysis of PDENs using GC-MS

**Fig 7:**
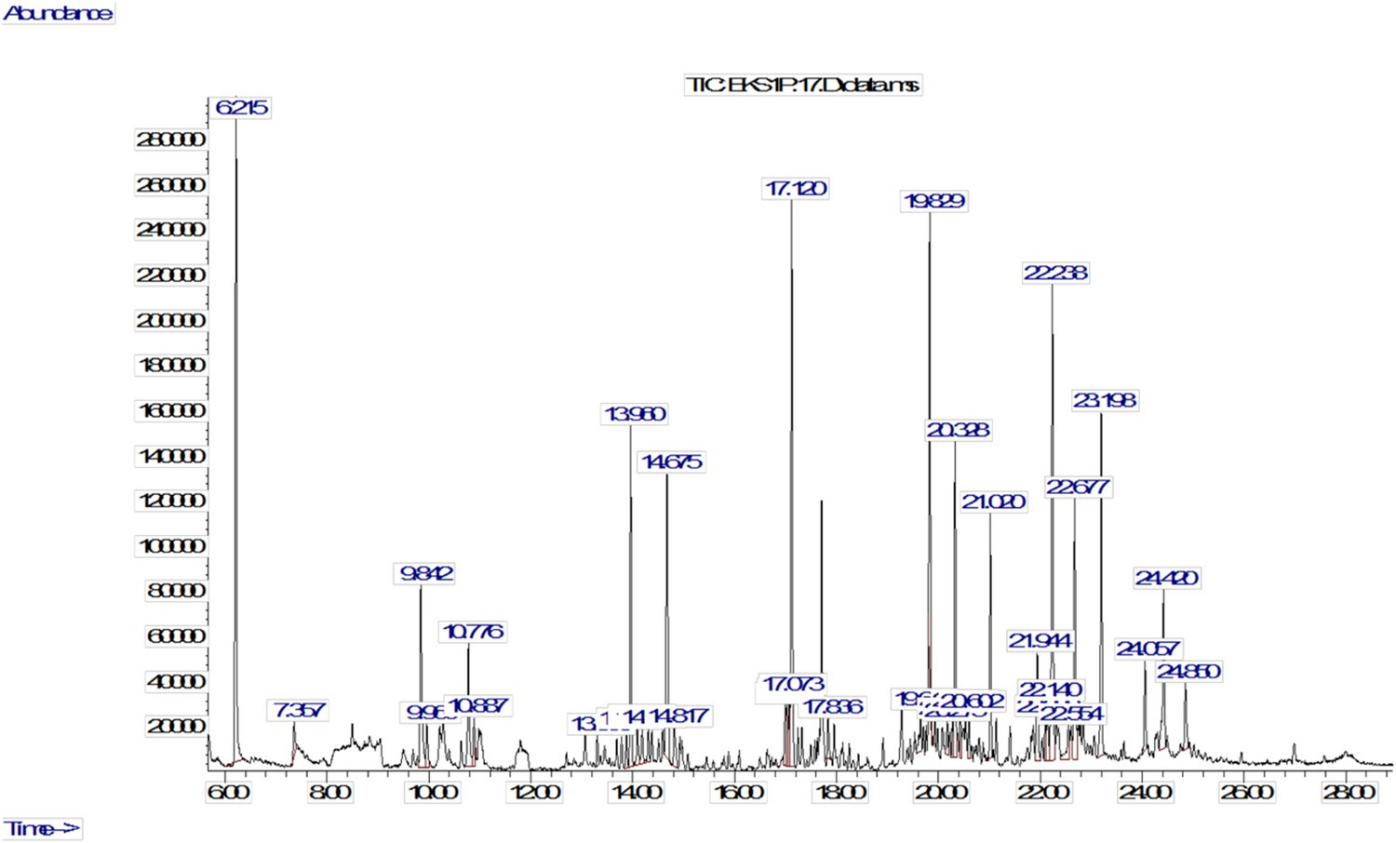
The GC-MS chromatogram of PDENs isolated from *Solanum nigrum* L. berries. The content of PDENs from *Solanum nigrum* L. berries was analyzed using GC-MS, showing the presence of lipid derivatives with long-chain structures.

GC-MS was performed to analyze the composition of PDENs that may contribute to their anti-inflammatory properties. The results indicated that the compounds present in PDENs are predominantly lipid derivatives with long-chain structures (**Fig 7**). There are 14 compounds with abundances above 2%, with seven of them having abundances above 5% as listed in **Table 1**. These include the compound 2-Amino-5-methylbenzoic acid, which has the highest abundance (11.68%), Eicosane (9.55%), Heneicosane (9.27%), 5(2) methylpropyl nonane (6.09%), Palmitaldehyde (5.9%), Tricosane (5.4%), and Pentacosane (5.26%).

**Table 1:** Identified molecules based-on GC-MS analysis on PDENs isolated from *Solanum nigrum* L. berries.

No.	Retention time (min)	Compounds	Formula	Molecular Weight (g/mol)	Area%
**1**	6.2147	2-Amino-5-methylbenzoic acid	C_8_H_9_NO_2_	151.16	11,68*
**2**	7.3570	2-Anthracenamine	C_14_H_11_N	193.24	0,58
**3**	9.8418	Hexadecane	C_16_H_34_	226.44	4,06*
**4**	9.9603	4,7-Dimethylundecane	C_13_H_28_	184.36	0,88
**5**	10.7759	3,6 dimethyldecane	C_12_H_26_	170.33	2,67*
**6**	10.8867	Nonadecane	C_19_H_40_	268.5	0,85
**7**	13.3056	Neral/Z citral	C_10_H_16_O	152.23	0,5
**8**	13.8759	Docosane	C_22_H_46_	310.6	0,61
**9**	13.9603	Eicosane	C_20_H_42_	282.5	5,36*
**10**	14.0944	Octadecane	C_18_H_38_	254.5	0,75
**11**	14.1882	Pentacosane	C_25_H_52_	352.7	0,69
**12**	14.3097	Pentacosane	C_25_H_52_	352.7	0,6
**13**	14.6747	5(2) methylpropyl nonane	C_13_H_28_	184.36	6,09*
**14**	14.8169	2,3,7 trimethyldecane	C_13_H_28_	184.36	0,99
**15**	17.0278	Heneicosane	C_21_H_44_	296.6	1,11
**16**	17.0728	Eicosane	C_20_H_42_	282.5	1,03
**17**	17.1203	Eicosane	C_20_H_42_	282.5	9,55*
**18**	17.8359	Octadecane	C_18_H_38_	254.5	0,62
**19**	19.6516	Heneicosane	C_21_H_44_	296.6	0,9
**20**	19.8291	Heneicosane	C_21_H_44_	296.6	6,19*
**21**	20.1755	2,6-Diisopropylnaphthalene	C_16_H_20_	212.33	0,57
**22**	20.2750	Octacosane	C_28_H_58_	394.8	0,5
**23**	20.3283	Tricosane	C_23_H_48_	324.6	5,4*
**24**	20.4345	Heneicosane	C_21_H_44_	296.6	0,74
**25**	20.6021	Docosane	C_22_H_46_	310.6	0,73
**26**	21.0199	Pentadecanal	C_15_H_30_O	226.40	3,59*
**27**	21.4073	Pentacosane	C_25_H_52_	352.7	2,09*
**28**	22.0991	Octadecane	C_18_H_38_	254.5	0,65
**29**	22.1398	Heptacosane	C_27_H_56_	380.7	1,17
**30**	22.2385	Heneicosane	C_21_H_44_	296.6	9,27*
**31**	22.5544	Octadecane	C_18_H_38_	254.5	1,02
**32**	22.6766	Pentacosane	C_25_H_52_	352.7	5,26*
**33**	23.1985	Palmitaldehyde	C_16_H_32_O	240.42	5,9*
**34**	24.0574	Stearonitrile	C_18_H_35_N	265.5	1,55
**35**	24.4199	Tricosane	C_23_H_48_	324.6	3,47*
**36**	24.8496	Eicosane	C_20_H_42_	282.5	1,4

*) *compounds with abundance above 2%*.

## Discussion

PDENs are an attractive candidate for cell-free therapy. PDENs are easier to obtain, have fewer safety concerns, and can be more efficiently produced compared to exosomes from mammalian cells [9]. PDENs can also regulate gene expression in a cross-kingdom manner [10]. Moreover, PDENs have been shown to have anti-inflammatory properties [11–13]. However, there are limited reported studies on PDENs and many plant sources of PDENs have yet to be investigated.

Black nightshade berries (*Solanum nigrum* L.) have a range of medicinal properties, including anti-inflammatory effects [17,18]. The present study therefore investigated the anti-inflammatory properties of PDENs derived from *Solanum nigrum* L. berries. PDENs were isolated using a PEG-based precipitation method previously described by Kalarikkal et al. (2020) [19] to isolate ginger-derived nanoparticles. PEG has widely been used to precipitate proteins, DNA, and viral particles [20–22]. In recent years, the PEG precipitation method has also been used to isolate nanovesicles, including exosomes [23]. PEG is a hydrophilic polymer with low toxicity that can be used to precipitate nanovesicles by low-speed centrifugation [24]. The PEG-based precipitation method has been reported to yield exosomes with similar characteristics compared to exosomes isolated using ultracentrifugation, which is the most widely used method for exosome isolation [19]. Furthermore, the PEG-based precipitation method has previously been observed to produce higher amounts of exosomes with higher exosomal DNA content compared to isolation methods using commercial kits, such as ExoQuick® and PureExo®[25]. These findings indicated that PEG-based precipitation can provide a simpler, cost-effective, and more efficient method of isolating exosomes than the use of commercial kits or ultracentrifugation.

In the present study, PDENs isolated from *Solanum nigrum* L. berries had spherical morphology, with an average size of 107 nm in keeping with the known size of exosome from previous studies [12,13,26]. The zeta potential was also measured to assess the stability of PDENs in suspension and their adsorption capacity on the target cell membrane [27]. PDENs in general have negative zeta potential that lies between -100 and 0 mV, which varies between different plant sources [28]. The total protein concentration of PDENs were measured using bicinchoninic acid assay (BCA Assay), which based on the reaction between Cu^+^ ion and sodium salt from bicinchoninic acid in an alkaline solution, producing a purple-colored solution that absorbs light at 562 nm [29]. The quantified protein in nanoparticles such as exosomes or PDENs is indirectly correlated with the number of particles [30] and can be affected by the mass and quality of the fruit during isolation [14].

The effect of storage temperature and duration on PDENs stability are important to consider for clinical application. In the current study, PDENs stored at 4°C and -20°C showed size fluctuation over four weeks of storage. Based on the result, a preservative might be required to maintain the stability of PDENs through repeated freeze-thaw cycle. Previous study reported that plant leaf-derived extracellular vesicles had greater stability at 4°C when coupled with TMO, a preservative developed for the cosmetic industry [31]. More research on the stability of PDENs are highly suggested for future application.

The uptake of PDENs into cells is an important process for drug delivery and therapeutic efficacy. The internalization of PDENs in mammalian cells is known to occur through several mechanisms, such as plasma membrane fusion [32], clathrin-dependent endocytosis, and macropinocytosis [33]. Moreover, it is also known that extracellular vesicles can be internalized through phagocytosis by phagocytic cells [34], hence it is quite likely that PDENs in this study were internalized through phagocytosis by RAW264.7 cells, which are macrophage cells line. The uptake of PDENs can be influenced by the plant sources, target cells, and the lipid content of the membrane [35–37]. PDENs have differing lipid compositions to animal cells. The membrane of PDENs is composed of phospholipids, including phosphatidic acid (PA), phosphatidylcholines, digalactosyldiacylglycerol, and monogalactosyldiacylglycerol [9]. Of these, PA is known to facilitate membrane fusion and fission and may contribute to the internalization of PDENs into animal cells [36,37]. The results of the present study demonstrate that PDENs are taken up by a mouse macrophage cell line, RAW264.7, within two hours of incubation.

The cytotoxicity of PDENs in their target cells is important to be investigated. Some PDENs are expected to exhibit potent cytotoxicity toward tumor or cancer cells [38,39]. In the present study, PDENs will be used as a therapeutic agent for macrophage cells, which modulate the inflammatory process. Accordingly, PDENs must be safe and non-toxic to these cells. PDENs had a toxicity limit on RAW264.7 cells up to a concentration of 2.5 μg/mL. Higher concentrations of PDENs (≥ 5 μg/mL) significantly decreased RAW264.7 cells viability to as low as 4%, which may be due to the presence of certain compounds contained in PDENs. The cytotoxicity of PDENs is strongly influenced by their source. For instance, PDENs from lemon are not toxic to RAW264.7 cells up to a concentration of 50 μg/mL [14], while PDENs from grapefruit showed no cytotoxicity up to a concentration of 60 μg/mL [33]. *Solanum nigrum* L. belongs to the *Solanaceae* family, which is known to contain glycoalkaloid compounds such as solanine. These compounds are known to be toxic when consumed in excess [16]. Therefore, PDENs with a concentration more than 2.5 μg/mL will not be used in subsequent experiments.

**Fig 8:**
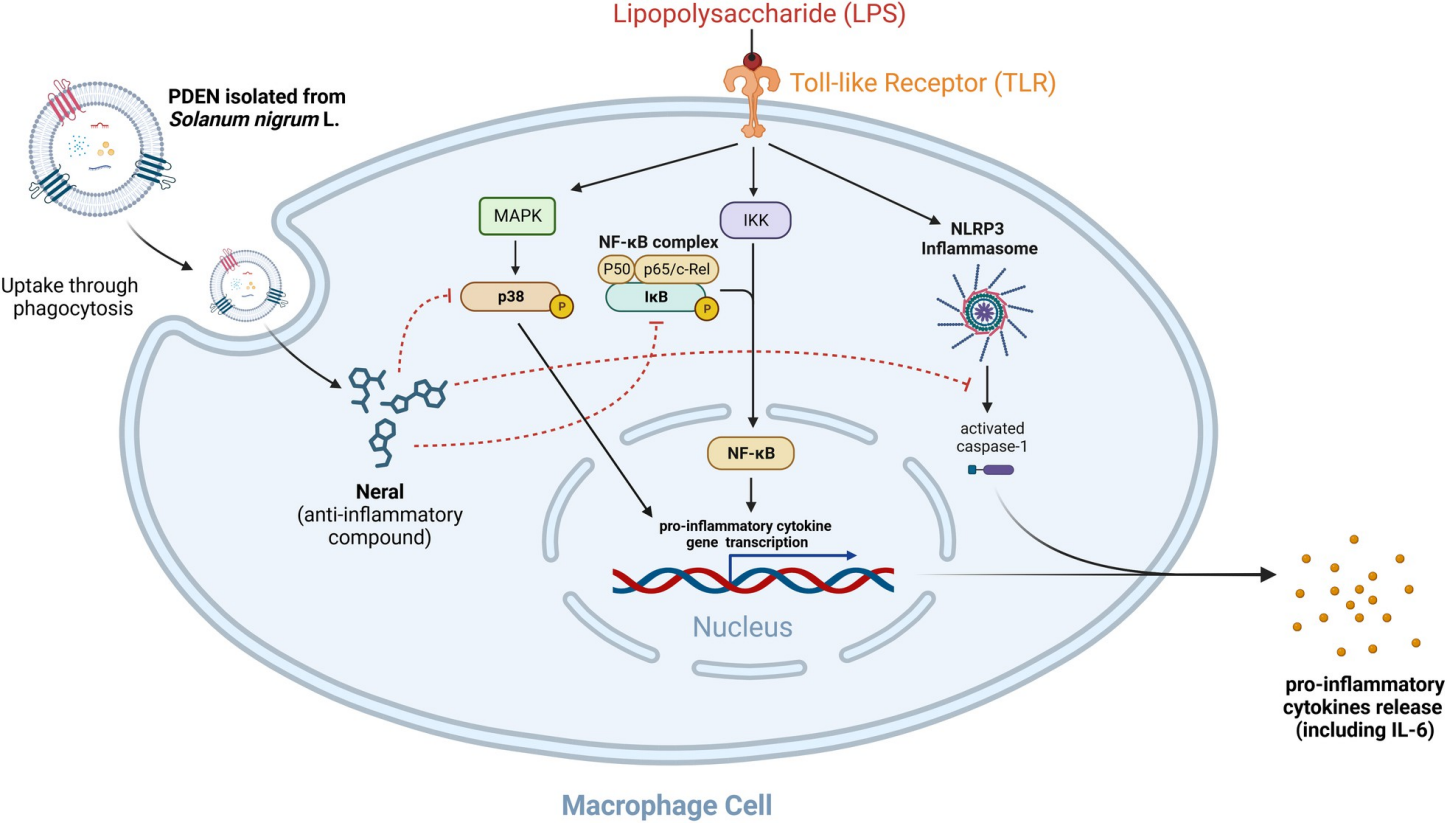
Hypothetical Anti-Inflammatory Effects of PDENs Isolated from *Solanum nigrum* L. Berries in LPS-induced RAW264.7 Cell. PDENs isolated from *Solanum nigrum* L. berries were phagocytosed into the LPS-stimulated macrophage cells. PDENs contain various bioactive compounds, including neral, which is known to have potent anti-inflammatory effects by inhibiting p38 & IκB phosphorylation and activation of NLRP-3 inflammasome, preventing the release of pro-inflammatory cytokines including IL-6.

The present study also investigated the anti-inflammatory effects of PDENs in LPS-stimulated RAW264.7 cells. LPS can induce polarization toward M1 macrophages and activate the NF-κB pathway to induce the expression of pro-inflammatory cytokine genes. Activated macrophages secrete pro-inflammatory cytokines, such as interleukin-6 (IL-6), as a defense mechanism [40]. In the present study, PDENs treatment suppressed IL-6 gene expression and protein secretion by LPS-stimulated RAW264.7 cells. At the concentration of 1 μg/mL, PDENs had a greater anti-inflammatory effect than dexamethasone, a common anti-inflammatory drug. Furthermore, the addition of PDENs at a concentration of 2.5 μg/mL showed the greatest effect in attenuating the gene expression and protein secretion of IL-6 by RAW264.7 cells in response to LPS. The anti-inflammatory activity of PDENs is known to be influenced by the bioactive compound present within the nanoparticles [13,14]. GC-MS content analysis revealed that PDENs contain a variety of compounds that are identified as natural substances found in plants and display specific bioactivities. Among these, 2-amino-5-methylbenzoic acid, a derivative of anthranilic acid, has been reported to have potent anti-tumor activities and inhibits cancer cell development [41,42]. Furthermore, this compound has the highest abundance (11.68%), indicating that PDENs derived from *Solanum nigrum* L. berries may have significant anti-tumor or anti-cancer properties that can be investigated further in future studies. The results also showed the presence of neral, which is a Z-isomer form of citral, a monoterpene typically found in lemons that has anti-inflammatory properties [43,44]. Previous study showed that neral extracted from fruits of *Litsea cubeba* have potent anti-inflammatory activity in LPS-induced macrophage cells by inhibiting the activation of NLRP-3 inflammasome and phosphorylation of p38 & IκB, which will prevent the transcription of pro-inflammatory cytokine genes including IL-6 [45]. This finding demonstrates that, despite its low concentration (0.5%), the compound neral may have a considerable impact on the anti-inflammatory properties of PDENs isolated from *Solanum nigrum* L. berries. However, the underlying mechanisms on the anti-inflammatory effects of neral from PDENs have not been investigated in this study. We propose here the hypothetical mechanism for the observed anti-inflammatory effects of PDENs isolated from *Solanum nigrum* L. berries (**Fig 8**).

The present study is the first to successfully isolate and characterize PDENs from *Solanum nigrum* L. berries (PDENs). Isolated PDENs had a spherical morphology with an average size distribution of 107 nm and zeta potential of -0.6 mV. PDENs were shown to be internalized by macrophage cells and had no cytotoxicity effect up to the concentration of 2.5 μg/mL. The results of the present study also demonstrate the anti-inflammatory activity of PDENs isolated from *Solanum nigrum* L. berries by suppressing the production of IL-6 in LPS-stimulated RAW264.7 cells. GC-MS analysis showed the presence of neral, a monoterpene compound with known anti-inflammatory properties, which may contribute to the anti-inflammatory activity of PDENs isolated from *Solanum nigrum* L. berries. These findings indicate PDENs isolated from *Solanum nigrum* L. berries may have utility in the treatment of inflammation-related diseases.

## Supporting information

S1 File (ZIP)

## Abbreviations

PDEN: Plant-derived exosome-like nanoparticle
DMEM: Dulbecco’s modified eagle medium
PEG: Polyethylene glycol
NTA: Nanoparticle Tracking Analysis
DLS: Dynamic light scattering
TEM: Transmission electron microscopy
BCA: Bicinchoninic acid
ELISA: Enzyme-linked immunosorbent assay
RT-qPCR: Quantitative reverse transcription polymerase chain reaction
IL-6: Interleukin-6
GAPDH: Glyceraldehyde 3-phosphate dehydrogenase
LPS: Lipopolysaccharide
GC-MS: Gas Chromatography-Mass Spectrometry
NF-κB: Nuclear Factor Kappa-B

## References

[1] Furman D, Campisi J, Verdin E, Carrera-Bastos P, Targ S, Franceschi C, et al. Chronic inflammation in the etiology of disease across the life span. Nat Med. 2019;25: 1822–1832. doi: 10.1038/s41591-019-0675-0 31806905 PMC7147972

[2] Fleit HB. Chronic Inflammation. In: McManus LM, Mitchell RNBT-P of HD, editors. San Diego: Academic Press; 2014. pp. 300–314. 10.1016/B978-0-12-386456-7.01808-6.

[3] Marcum ZA, Hanlon JT. Recognizing the Risks of Chronic Nonsteroidal Anti-Inflammatory Drug Use in Older Adults. 2010. PMC3158445 21857795

[4] Volmer T, Effenberger T, Trautner C, Buhl R. Consequences of long-term oral corticosteroid therapy and its side-effects in severe asthma in adults: A focused review of the impact data in the literature. European Respiratory Journal. European Respiratory Society; 2018. doi: 10.1183/13993003.00703–2018 30190274

[5] Kalluri R, LeBleu VS. The biology, function, and biomedical applications of exosomes. Science. 2020;367. doi: 10.1126/science.aau6977 32029601 PMC7717626

[6] Bunggulawa EJ, Wang W, Yin T, Wang N, Durkan C, Wang Y, et al. Recent advancements in the use of exosomes as drug delivery systems. J Nanobiotechnology. 2018;16: 81. doi: 10.1186/s12951-018-0403-9 30326899 PMC6190562

[7] Suh JH, Joo HS, Hong EB, Lee HJ, Lee JM. Therapeutic application of exosomes in inflammatory diseases. Int J Mol Sci. 2021;22: 1–22. doi: 10.3390/ijms22031144 33498928 PMC7865921

[8] Wang C, Xu M, Fan Q, Li C, Zhou X. Therapeutic potential of exosome-based personalized delivery platform in chronic inflammatory diseases. Asian J Pharm Sci. 2022; 100772. doi: 10.1016/j.ajps.2022.100772 36896446 PMC9989662

[9] Kim J, Li S, Zhang S, Wang J. Plant-derived exosome-like nanoparticles and their therapeutic activities. Asian J Pharm Sci. 2022;17: 53–69. doi: 10.1016/j.ajps.2021.05.006 35261644 PMC8888139

[10] Xiao J, Feng S, Wang X, Long K, Luo Y, Wang Y, et al. Identification of exosome-like nanoparticle-derived microRNAs from 11 edible fruits and vegetables. PeerJ. 2018;6: e5186. doi: 10.7717/peerj.5186 30083436 PMC6074755

[11] Zhang M, Xiao B, Wang H, Han MK, Zhang Z, Viennois E, et al. Edible Ginger-derived Nano-lipids Loaded with Doxorubicin as a Novel Drug-delivery Approach for Colon Cancer Therapy. Molecular Therapy. 2016;24: 1783–1796. doi: 10.1038/mt.2016.159 27491931 PMC5112046

[12] You JY, Kang SJ, Rhee WJ. Isolation of cabbage exosome-like nanovesicles and investigation of their biological activities in human cells. Bioact Mater. 2021;6: 4321–4332. doi: 10.1016/j.bioactmat.2021.04.023 33997509 PMC8105599

[13] Kang SJ, Kim SE, Seo M-J, Kim E, Rhee WJ. Suppression of inflammatory responses in macrophages by onion-derived extracellular vesicles. Journal of Industrial and Engineering Chemistry. 2022;115: 287–297. 10.1016/j.jiec.2022.08.011.

[14] Raimondo S, Urzì O, Meraviglia S, Di Simone M, Corsale AM, Rabienezhad Ganji N, et al. Anti‐inflammatory properties of lemon‐derived extracellular vesicles are achieved through the inhibition of ERK/NF‐κB signalling pathways. J Cell Mol Med. 2022;26: 4195–4209. doi: 10.1111/jcmm.17404 35789531 PMC9344827

[15] Chen X, Dai X, Liu Y, Yang Y, Yuan L, He X, et al. Solanum nigrum Linn.: An Insight into Current Research on Traditional Uses, Phytochemistry, and Pharmacology. Front Pharmacol. 2022;13. doi: 10.3389/fphar.2022.918071 36052142 PMC9424827

[16] Kuete V. 22—Physical, Hematological, and Histopathological Signs of Toxicity Induced by African Medicinal Plants. In: Kuete VBT-TS of AMP, editor. Elsevier; 2014. pp. 635–657. 10.1016/B978-0-12-800018-2.00022-4.

[17] Ravi V, Sheikuduman MS, Patel S, Raamamurthy J, Karunakaran G. Anti-Inflammatory Effect of Methanolic Extract of Solanum nigrum Linn Berries. International Journal of Applied Research in Natural Products. 2009;2.

[18] Wang Y, Xiang L, Yi X, He X. Potential Anti-inflammatory Steroidal Saponins from the Berries of Solanum nigrum L. (European Black Nightshade). J Agric Food Chem. 2017;65: 4262–4272. doi: 10.1021/acs.jafc.7b00985 28486801

[19] Kalarikkal SP, Prasad D, Kasiappan R, Chaudhari SR, Sundaram GM. A cost-effective polyethylene glycol-based method for the isolation of functional edible nanoparticles from ginger rhizomes. Sci Rep. 2020;10: 4456. doi: 10.1038/s41598-020-61358-8 32157137 PMC7064537

[20] He Z, Zhu Y, Gu H. A new method for the determination of critical polyethylene glycol concentration for selective precipitation of DNA fragments. Appl Microbiol Biotechnol. 2013;97: 9175–9183. doi: 10.1007/s00253-013-5195-0 23982329

[21] Ingham KC. [23] Precipitation of proteins with polyethylene glycol. In: Deutscher MP, editor. Methods in Enzymology. Academic Press; 1990. pp. 301–306. 10.1016/0076-6879(90)82025-W. 2314243

[22] Lewist GD, Metcalf TG. Polyethylene Glycol Precipitation for Recovery of Pathogenic Viruses, Including Hepatitis A Virus and Human Rotavirus, from Oyster, Water, and Sediment Samples. Appl Environ Microbiol. 1988. Available: https://journals.asm.org/journal/aem. 10.1128/aem.54.8.1983-1988.1988 PMC202790 2845860

[23] Weng Y, Sui Z, Shan Y, Hu Y, Chen Y, Zhang L, et al. Effective isolation of exosomes with polyethylene glycol from cell culture supernatant for in-depth proteome profiling. Analyst. 2016;141: 4640–4646. doi: 10.1039/c6an00892e 27229443

[24] Sidhom K, Obi PO, Saleem A. A review of exosomal isolation methods: Is size exclusion chromatography the best option? International Journal of Molecular Sciences. MDPI AG; 2020. pp. 1–19. doi: 10.3390/ijms21186466 32899828 PMC7556044

[25] García-Romero N, Madurga R, Rackov G, Palacín-Aliana I, Núñez-Torres R, Asensi-Puig A, et al. Polyethylene glycol improves current methods for circulating extracellular vesicle-derived DNA isolation. J Transl Med. 2019;17: 75. doi: 10.1186/s12967-019-1825-3 30871557 PMC6419425

[26] Kim J, Lee Y-H, Wang J, Kim YK, Kwon IK. Isolation and characterization of ginseng-derived exosome-like nanoparticles with sucrose cushioning followed by ultracentrifugation. SN Appl Sci. 2022;4: 63. doi: 10.1007/s42452-022-04943-y

[27] Rasmussen MK, Pedersen JN, Marie R. Size and surface charge characterization of nanoparticles with a salt gradient. Nat Commun. 2020;11: 2337. doi: 10.1038/s41467-020-15889-3 32393750 PMC7214416

[28] Li A, Li D, Gu Y, Liu R, Tang X, Zhao Y, et al. Plant-derived nanovesicles: Further exploration of biomedical function and application potential. Acta Pharm Sin B. 2023;13: 3300–3320. doi: 10.1016/j.apsb.2022.12.022 37655320 PMC10465964

[29] Shen C-H. Quantification and Analysis of Proteins. Diagnostic Molecular Biology. Elsevier; 2019. pp. 187–214. doi: 10.1016/B978-0-12-802823-0.00008–0

[30] Dash M, Palaniyandi K, Ramalingam S, Sahabudeen S, Raja NS. Exosomes isolated from two different cell lines using three different isolation techniques show variation in physical and molecular characteristics. Biochimica et Biophysica Acta (BBA)—Biomembranes. 2021;1863: 183490. doi: 10.1016/j.bbamem.2020.183490 33212036

[31] Kim K, Park J, Sohn Y, Oh C-E, Park J-H, Yuk J-M, et al. Stability of Plant Leaf-Derived Extracellular Vesicles According to Preservative and Storage Temperature. Pharmaceutics. 2022;14: 457. doi: 10.3390/pharmaceutics14020457 35214189 PMC8879201

[32] Subha D, Harshnii K, Madhikiruba KG, Nandhini M, Tamilselvi KS. Plant derived exosome- like Nanovesicles: an updated overview. Plant Nano Biology. 2023;3: 100022. doi: 10.1016/j.plana.2022.100022

[33] Wang B, Zhuang X, Deng Z-B, Jiang H, Mu J, Wang Q, et al. Targeted Drug Delivery to Intestinal Macrophages by Bioactive Nanovesicles Released from Grapefruit. Molecular Therapy. 2014;22: 522–534. doi: 10.1038/mt.2013.190 23939022 PMC3944329

[34] Feng D, Zhao W-L, Ye Y-Y, Bai X-C, Liu R-Q, Chang L-F, et al. Cellular Internalization of Exosomes Occurs Through Phagocytosis. Traffic. 2010;11: 675–687. doi: 10.1111/j.1600-0854.2010.01041.x 20136776

[35] Yi Q, Xu Z, Thakur A, Zhang K, Liang Q, Liu Y, et al. Current understanding of plant-derived exosome-like nanoparticles in regulating the inflammatory response and immune system microenvironment. Pharmacol Res. 2023;190: 106733. doi: 10.1016/j.phrs.2023.106733 36931541

[36] Shkryl Y, Tsydeneshieva Z, Degtyarenko A, Yugay Y, Balabanova L, Rusapetova T, et al. Plant Exosomal Vesicles: Perspective Information Nanocarriers in Biomedicine. Applied Sciences (Switzerland). MDPI; 2022. doi: 10.3390/app12168262

[37] Zhukovsky MA, Filograna A, Luini A, Corda D, Valente C. Phosphatidic acid in membrane rearrangements. FEBS Lett. 2019;593: 2428–2451. doi: 10.1002/1873-3468.13563 31365767

[38] Chen Q, Li Q, Liang Y, Zu M, Chen N, Canup BSB, et al. Natural exosome-like nanovesicles from edible tea flowers suppress metastatic breast cancer via ROS generation and microbiota modulation. Acta Pharm Sin B. 2022;12: 907–923. doi: 10.1016/j.apsb.2021.08.016 35256954 PMC8897038

[39] Raimondo S, Naselli F, Fontana S, Monteleone F, Lo Dico A, Saieva L, et al. Citrus limon-derived nanovesicles inhibit cancer cell proliferation and suppress CML xenograft growth by inducing TRAIL-mediated cell death. Oncotarget. 2015;6: 19514–19527. doi: 10.18632/oncotarget.4004 26098775 PMC4637302

[40] Liu T, Zhang L, Joo D, Sun S-C. NF-κB signaling in inflammation. Signal Transduct Target Ther. 2017/07/14. 2017;2: 17023. doi: 10.1038/sigtrans.2017.23 29158945 PMC5661633

[41] Purushottamachar P, Khandelwal A, Vasaitis TS, Bruno RD, Gediya LK, Njar VCO. Potent anti-prostate cancer agents derived from a novel androgen receptor down-regulating agent. Bioorg Med Chem. 2008;16: 3519–3529. doi: 10.1016/j.bmc.2008.02.031 18316193

[42] Abuelizz HA, Marzouk M, Ghabbour H, Al-Salahi R. Synthesis and anticancer activity of new quinazoline derivatives. Saudi Pharmaceutical Journal. 2017;25: 1047–1054. doi: 10.1016/j.jsps.2017.04.022 29158714 PMC5681317

[43] Bouzenna H, Hfaiedh N, Giroux-Metges M-A, Elfeki A, Talarmin H. Biological properties of citral and its potential protective effects against cytotoxicity caused by aspirin in the IEC-6 cells. Biomedicine & Pharmacotherapy. 2017;87: 653–660. doi: 10.1016/j.biopha.2016.12.104 28088731

[44] Amorim JL, Simas DLR, Pinheiro MMG, Moreno DSA, Alviano CS, da Silva AJR, et al. Anti-Inflammatory Properties and Chemical Characterization of the Essential Oils of Four Citrus Species. PLoS One. 2016;11: e0153643. doi: 10.1371/journal.pone.0153643 27088973 PMC4835072

[45] Liao P-C, Yang T-S, Chou J-C, Chen J, Lee S-C, Kuo Y-H, et al. Anti-inflammatory activity of neral and geranial isolated from fruits of Litsea cubeba Lour. J Funct Foods. 2015;19: 248–258. doi: 10.1016/j.jff.2015.09.034

